# What is quantum biology?

**DOI:** 10.1073/pnas.2531134123

**Published:** 2026-03-20

**Authors:** Gregory D. Scholes, Graham R. Fleming

**Affiliations:** ^a^Department of Chemistry, Princeton University, Princeton, NJ 08544; ^b^Department of Chemistry and QB3 Institute, University of California, Berkeley, CA 94720; ^c^Molecular Biophysics and Integrated Bioimaging Division, Lawrence Berkeley National Laboratory, Berkeley, CA 94720

**Keywords:** quantum biology, coherence, quantum probes, quantum mimics

## Abstract

Quantum biology is the field at the intersection of quantum-related physics and the biology of living systems. The goal of the field is to determine if quantum phenomena underpin biological function at the macroscale. Such results, supported by compelling experimental evidence, will be important because they will show how quantum effects can have functional relevance, even in very complex and nominally classical systems. Here, we attempt to define the scope of quantum biology with a forward-facing view to help focus the research agenda. To that end, we propose open questions fundamental to consolidating the field of quantum biology. These open questions highlight the importance of developing suitable probes at the quantum scale, the possibility that classical biological machinery might simply mimic quantum systems, and of elucidating the ways quantum function can be amplified to the macroscale.

Quantum biology sounds implausible. When examining living organisms, we encounter molecules in warm, wet, and noisy environments. Such environments are the very antithesis of what is usually required for true quantum effects such as entanglement and coherence arising from superpositions to exist. It is this paradox that motivates the search for quantum-mechanical phenomena giving rise to functional advantages for living systems (1–9).

At the molecular scale in biology, we can indeed find “traditional” quantum effects. For instance, a key element of quantum-mechanics is the wavelike basis for matter. This feature comes into prominence in biological systems when electrons or protons are involved in biochemical processes, and this provides a compelling molecular-scale example of quantum mechanics in biology. For instance, systematic comparisons of the rates of reaction for certain classes of enzymes, like hydrogenases, show that the wavelike properties of protons must be engaged in the enzyme active sites (10). The evidence is collected by comparing the reaction rates catalyzed by those enzymes when hydrogen versus deuterium is being transferred. The de Broglie wavelength of deuterium is smaller than that of hydrogen, so it is “less wavelike.” Rates of reaction of hydrogen-containing substrates can be orders of magnitude higher than those for deuterium, suggesting that the activation barrier for the reaction is circumvented by wavelike hydrogen leaking through it—a mechanism known as tunneling.

The promise of quantum biology, however, is identifying a significantly expanded scope of quantum phenomena. Let us begin our definition of quantum biology by stating what it is not: It is not a grab bag for all biological phenomena that lack a full quantitative understanding at present. Perhaps a clue toward defining quantum biology lies in the parallel tracks of two investigations that have raised questions concerning quantum effects in biological function. These are studies of the primary steps of photosynthesis, especially light harvesting, and migratory bird navigation. Each of these biological functions, despite operating on wildly differing timescales, raised questions about the role of quantum mechanics that have proved remarkably difficult to answer definitively. These advances would not have been possible until the development of the relevant measurement methods.

The seeds for each of these areas were sown in the late 1960s. In his only paper on photosynthesis, G W Robinson described “exciton spreading” occurring as fast as 50 fs in light harvesting systems (11). All previous discussions of photosynthetic energy transfer referred to “hopping” of excitation, rather than the wave-like picture implied by exciton spreading. In parallel, Wolfgang Wiltschko studied migrating northern European robins and showed that, when exposed to an artificial magnetic field of similar strength to the Earth’s magnetic field, the birds could orient themselves correctly—even when in a windowless room where they could see neither sun nor stars (12). When the direction of the magnetic field was changed, so did the birds’ orientation.

In this article, we attempt to define the scope of quantum biology with a forward-facing view that will help focus its agenda as a field. We aim to suggest how to approach the development of the field and where the key advances may lie. To that end, we propose some open questions that appear to be important for moving the field forward. If quantum effects can be shown to benefit biological systems, this completely changes our view on how the quantum world can impact the macroscale. Indeed, in this case, an understanding of the role of quantum effects in specific biological systems is necessary to fully delineate the design principles used by the natural system. Therefore, a deeper understanding of how such effects could be manifest in biosystems, and better ways of measuring these effects, are vital to truly understanding biological phenomena of all sorts. There are many fields that would stand to gain from such advances.

## Defining Quantum Biology

### A Sweeping Definition of Quantum Biology Is That It Is the Identification and Study of Quantum Phenomena in Biological Systems.

It is a subset of the broader concept of quantum-related photobiology that encompasses light-induced processes, magnetic field effects that are not necessarily quantum-mechanical, and transformations involving quantum particles (electrons, protons, photons). In recent years, quantum biology has begun to influence other fields, such as quantum simulation (13–15), suggesting that now is a good time to examine the question that is the title of this paper. We hope to inspire new directions.

Why is quantum biology difficult to define? When we consider physical phenomena, we usually silo them into a classical or a quantum model. Quantum biology challenges us because it breaks this rule, because we need to elucidate how the remarkably complex, yet sophisticated, machinery of life could recruit special correlations or transformations that appear to be unique to quantum systems with their wavelike nature. Quantum mechanics is generally framed as a theory about states and their linear transformations, whereas biology concerns structure and function and complex nonlinear machinery. What lies at this interface?

Quantum biology is at present dominated by a search for functional quantum mechanics hidden in complex biosystems. Discovering concrete and compelling examples is crucial. During this search, we can be faced with extremely difficult and, sometimes, ephemeral questions; a quest for phenomena and mechanisms that we are not even certain to exist. It is therefore pressing that we define pathways forward that enable the development of testable hypotheses and relevant new experiments. In other words, quantum biology often includes the identification and study of phenomena that are not yet satisfactorily explained.

Famously, mesmerism caused great excitement in the late 18^th^ century by the claim of a miraculously restorative “universal magnetic fluid” that was, nevertheless, undetectable by scientific methods (16). In quantum biology, we need to be careful to avoid choosing our observations to provide evidence supporting the phenomena we want to find. That can be avoided by posing well-framed open questions.

Examples of issues to be tackled include: a) What undiscovered functions could be enabled by quantum effects in biological systems? b) What are the mechanisms and what are the crucial quantum ingredients? c) Are these mechanisms plausible? d) Can we articulate an advantage that the quantum mechanism confers compared to a classical counterpart? e) Are there ways in which a large classical system can appear to exhibit quantum properties? f) What experiments are needed to address the proposed models and hypotheses?

## Concepts and Observations

In this article, we will not attempt to review the field of quantum biology; instead, we will collect ideas that we hope will help illuminate future progress. Our aim in this section is to provide some selected examples that give the reader a sense of some work in the field and that emphasize relevant concepts, and that suggest ways to think about quantum biology. With this discussion in mind, at the end of the paper, we will return to the question: “What is quantum biology?” and suggest some forward-looking answers.

### Electromagnetic Fields in Biology.

The diversity and sophistication of biology is astounding. Not only is intricate machinery assembled to order and used synergistically with hundreds of other functional systems, but seemingly disparate systems work in harmony on many length scales and across compartmentalized heterogeneity. However, it is still remarkable to realize that biological systems have developed the capability of engaging with fields.

We know a lot about how biological systems interact with light. Light prominently features in biology via photosynthesis, vision, photoprotective systems, photobiological synthesis of vitamins, phototaxis, and so on. Hundreds of proteins have been identified that use light for signaling and regulation (17–19), for example, activating biosynthetic pathways or enabling cellular cycles to be entrained to day-night periods—circadian rhythms. There are also many examples where biological systems control light using photonics (20). This may simply be as a means of attaining remarkable color, like in the cases of butterfly wings or bird feathers, or there could be functional adaptations in response to environment, often associated with organelles called iridocytes. For example, photonic structures in begonia leaves are produced in response to low ambient light conditions (21). Special organelles in giant clams increase the depth of light penetration into tissue, thus enhancing the growth of symbiotic microalgae (22).

Photobiologists have also conjectured that biologically sourced light, for example, emitted by reactive oxygen species, is harnessed for signaling between cells (23) and that light can be used in medical treatments, highlighted by photobiomodulation therapy (24). While these select examples illustrate some of the diverse ways biology uses light, uniquely quantum mechanisms are not necessarily involved. Nevertheless, this is a rich topic for exploration.

The interaction and exploitation of magnetic fields by biological systems is intriguing (25). Here, what is known is more sparse, likely because researchers are still developing relevant experimental probes. The avian navigation already mentioned is a good example of that point: Pinpointing the molecular mechanism behind magnetic field sensing has so far been a difficult challenge. However, there are now increasing examples that indicate radical pairs may be quite widely exploited in biological systems for sensors (26).

It therefore makes sense to claim that an obvious candidate for defining quantum biology is to include functional systems that use or are activated by fields. We suggest that the interaction of biological systems with fields, including optical, electrical (27), and magnetic sources, provides the predominant examples of function in quantum biology.

### Coherence, Synchronization, and Resonance.

One well-studied example of how a biological system engages light for function is photosynthesis. Here, the concepts of coherence and resonance have been at the forefront of physical models explaining details of the underlying mechanisms. In photosynthesis, the vast majority of light-absorbing molecules (“chromophores,” including chlorophylls) are used to absorb incident light and direct the excitation energy to the photosystems—specialized enzymes that use the large energies localized in photoexcitations to perform difficult chemical reactions such as water splitting and carbon dioxide reduction (28). This light-harvesting process is important to amplify the rate of photoexcitation by sunlight so that each enzyme (photosystem) can be activated with the energy of four sequential excitations and not suffer from detrimental back-reactions.

We have learned much about the physical design principles underlying photosynthetic light-harvesting (29). Fundamentally, we know that the rapid and efficient transfer of excitation energy often happens by excitation “hopping” from one molecule to another—a kind of random walk (30). We also know that chromophores can crowd together in light-harvesting complexes (sometimes approaching to within 3 to 4 Å) so that some of the chromophores collectively capture and transfer excitation via quantum-mechanical molecular exciton states (31), which is where energy evolves in a wavelike fashion as mentioned in the introduction.

With these models in mind, it is remarkable that light-harvesting is so efficient given that excitations execute, on average, well over a thousand steps after initial absorption, yet still reach the photosystems with close to unit efficiency. Researchers thus wondered if coherence was modifying the dynamics of the process on an ultrafast timescale. That question was examined in detail after the development of experiments that could provide the requisite insights—two-dimensional electronic spectroscopy (32–34). A detailed account of these developments is given in Ref. (35). The net result is that coherence contributes, but in a subtle way. We require more sophisticated theoretical models for the dynamics than those that predict incoherent hopping of excitation (36) because G W Robinson was partly correct; the energy does not simply hop incoherently from molecule to molecule. However, the outcome is that the dynamics seems to be optimized rather than profoundly sped up, although this issue is not fully settled.

A relevant and fascinating observation is that the light-harvesting complexes have evolved to ensure that electronic energy gaps—energy differences that funnel excitation vectorially—match closely with vibrational energy gaps. This can be thought of as a coherent amplification of energy-conserving resonances, which is enabled by quantum-mechanical mixing of excitations and vibrations (vibronic states and coherences). Evolution has tweaked the local protein environments of the chromophores to set up these resonances. Such selection by evolution provides compelling reasons to believe that optimization of frequency resonances has functional importance.

Coherence is a broad topic based on the properties enabled by waves or phases that are held in lock-step. Coherence has the potential to allow functions and phenomena that cannot be found in a purely stochastic, noisy system (37). Coherence can be classical or quantum-mechanical, and generally it is difficult to distinguish these cases (38). Perhaps, in many cases, it is unnecessary to make the distinction. Striking examples of coherence include interference phenomena involving electron transport pathways through molecules.

Classical coherence, closely related to synchronization (39), prevails in living systems—from the collective function of cells to circadian rhythms. The mechanism of light-harvesting in photosynthesis provides a prominent example of coherence on the protein-scale that has revitalized the field of quantum biology through experiments that reveal how coherence is leveraged in photosynthetic light-harvesting. Here, coherence comes in the currency of molecular excitons; collective electronic states delocalized over several molecules (28, 40). The idea is that an excited state molecule couples to a nearby ground state molecule by a resonant interaction between transition densities, often well-approximated as the resonant interaction between transition dipoles. The interaction produces new eigenstates for the electronic excited states that are coherently spread over two or more molecules.

### Macroscopic Coherence.

Fritz London was among the first to consider whether and how quantum mechanics could extend to the macroscale (41). He considered examples that include superconductivity and superfluids, like helium, which is famous for its near-zero viscosity and frictionless flow. He also thought of macromolecules as extended electronic oscillators. Interestingly, this view coincides with our contemporary understanding of conjugated polymers (42), some of which can show extraordinary exciton coherence lengths when free from disorder (43, 44).

Biology happens on length scales typically greater than a few nanometers. An open question in quantum biology, therefore, is whether relevant quantum phenomena exist with such very large length scales. Extraordinary wavelike position quantum superpositions have been discovered over the past decades, even for macromolecules including proteins (45). Yet, experiments suggest that special conditions of ultrafast timescales and small reorganization energies are needed for superpositions to play a functional role in chemical reaction dynamics (46). Biological function occurs in a special structured environment such as the interior of proteins, where it has been speculated that the interaction between substrates or chromophores can be controlled to some extent. Thus, the search for compelling examples of large-scale coherence phenomena in a biological setting is worthwhile, and crucial to support hypotheses of complex quantum functions involving macromolecules (47).

In the search for large-scale coherence in biology, studies have focused on proteins that are not normally considered to be functionally associated with light absorption. In particular, the remarkable patterns of aromatic amino acids—that absorb light in the near-ultraviolet—in ubiquitous structural proteins, such as microtubules, have aroused attention. These massive periodic structures that are integral for eukaryotic cytoskeletons have protein sequences that are surprisingly little changed from the last common eukaryotic ancestor. Does this suggest some unforeseen function? Their role in brain function and consciousness has been famously posited (48), but that hypothesis awaits experimental verification.

Superradiance is a collective phenomenon where many molecules radiate fluorescence synchronously. Interactions among the molecules stabilize collective, delocalized quantum mechanical states that are evidenced by their enhanced transition strength, and thus shorter radiative lifetimes. Recent studies have examined superradiance and ultraviolet energy transfer (akin to light-harvesting) in microtubules (49, 50). The excitation diffusion length is expected to be short because of the small absorption strength of aromatic amino acids, but measurements find excitation to migrate further than can be rationalized by standard theory.

### Noise has Benefits.

Fascinating questions are indicated by the results of a recent study of electrodynamic interactions between macromolecules (51). In particular, an intriguing very low frequency, 73 GHz, concentration-dependent mode was detected using terahertz spectroscopy to probe a solution of proteins. The observation is an example of a collective phenomenon emerging in a complex system. It is clearly an interprotein interaction because of the concentration dependence. These interactions that enable proteins to attract each other are termed electrodynamic to emphasize that they involve forces between oscillating charges that are weakly screened, even in high dielectric environments, and therefore they are surprisingly long-ranged (52). The detection of a collective motion of some kind is intriguing. In particular, how does this coherent oscillation, with a frequency of 1% of the thermal energy at ambient temperature, emerge from a disordered biological system? The following may provide a clue.

Noise is usually considered “bad” because it obscures signals and decoheres quantum superpositions. As famously proclaimed by Schrödinger, life is “warm, wet and noisy”, which is often the reasoning suggesting why quantum phenomena should not prevail in biology (53). However, a phenomenon called stochastic resonance turns this dogma on its head (54, 55). The theory explains, for example, how a noisy ambient electric field can, counterintuitively, enhance the efficacy of the paddle fish’s electroreceptor system. So, the fish are more adept at hunting prey that should be obscured by a noisy background (56). A somewhat related mechanism might be involved in mirror neuron systems (57). These neural circuits rapidly duplicate the signals of other circuits in the same brain or circuits of another party’s brain. This process of mentally mirroring the actions or expressions of others is observed in EEG traces as a remarkable displaced synchronization of the same measured brain activity. It has important functional and sociological consequences, for instance, as the basis for empathy and mother–infant interplay.

The theory of stochastic resonance explains many instances in biology, geophysics, and physics where a small signal is made evident by noise, rather than being hidden. It works by a synchronization phenomenon. A small periodic signal, such as a very weak oscillating electric field, entrains a dominating noise-assisted process. The prototypical example for the latter is thermally activated barrier crossing in a double-well potential. Owing to a nonlinear feedback process, the small periodic signal becomes amplified in the barrier crossing, which thus acquires a signature of the weak signal. Stochastic resonance might similarly provide a mechanism by which small quantum mechanical or coherence phenomena can be amplified in a noisy classical host environment.

Noise may also assist transport of excitation in a disordered energy landscape by a quantum ratchet mechanism (58). The energy resonance condition for excitation transfer in a light-harvesting complex might be improved by strongly coupling two molecules to form an exciton state, while a subsequent step might be improved if excitation is localized on one of these molecules. That can be realized in a dimer of strongly coupled molecules that, after gaining excitation, relaxes rapidly to a localized excitation that transfers to an acceptor. These kinds of excitonic ratchets can be configured in various ways, and they can provide a mechanism for attenuating dissipative back transfer.

### Quantum Biological Mechanisms Can be Hidden.

The complexity of a system may hide the mechanism as the basis for its function. Such a situation is likely the norm for quantum biology. In these situations, what we can learn about the underlying mechanism will depend critically on the incisiveness of our experiments and the development of innovative, verifiable hypotheses.

A consequence of this issue is illustrated by the idea of universality. Here, properties of a wide range of seemingly unrelated phenomena can be explained by a single (universal) model. For instance, processes that seem to occur randomly across many scales—earthquake size, periods of mass extinction, avalanches—can be understood by a common theory of self-organized criticality (59, 60).

In a celebrated address at the International Congress of Mathematicians, Madrid 2006, Percy Deift evidenced through examples how diverse mathematical and physical problems can all be resolved using a common model (61)—random matrix theory. The examples included a question about the card game patience sorting—specifically, on average how large does the table need to be if we play with some number *N* cards? Compare this to the problem of predicting bus arrival times at a stop in a city (e.g. Cuernavaca, Mexico) where there are no schedules and not even a municipal transit authority! Remarkably, both these problems, as well as many others, are amenable to the same analysis technique. An implication is, if we could only observe these problems through the lens of random matrix theory, then it would be difficult to tell how very different the basic mechanisms are from one another.

In sum, the idea is that our analysis process may not be able to distinguish, even if they appear microscopically quite distinct, the underlying mechanisms of a complex system. This will occur when the diverse mechanisms are all explained through the lens of a common theory or experimental analysis that serves as a good representation for the mechanisms.

### Quantum from the Bottom-Up.

Many of the most puzzling quantum phenomena are epitomized by entanglement. It is thought that any biological functions leveraging such correlations would be exquisite examples of quantum biology because they would have no counterpart in systems exhibiting only classical correlations.

Quantum-mechanical states are presented in the tensor product basis, meaning that the system is formed as a composite of building blocks, for instance, qubits, whose states live in their respective Hilbert spaces (i.e. coordinate systems defining the vectors). The tensor product basis means we index the composite system by products of the state of each Hilbert space, thus forming a completely new Hilbert space for the entire system. It turns out that certain superpositions of states in this basis cannot be factored into states local to any of the Hilbert spaces from which the basis is constructed. These are known as entangled states. A remarkable consequence of entanglement is that a measurement carried out on one qubit instantaneously determines the state of a second qubit, no matter how far apart the qubits are. This is the principle of nonlocality. See refs. 62, 63 for more detailed, but accessible, explanations.

It is, of course, tantalizing to imagine how a biological system might leverage such peculiar correlations. Therefore, the hypothesis (64) that bird navigation exploits entanglement among a pair of electrons separated within a cryptochrome protein has received much attention (65). The model assumes that once separated the pair of spins in the entangled singlet state can lose correlation, and this will partially block recombination by the Pauli principle. The extent to which correlations are lost when the spins are entangled depends on the direction of an external magnetic field, thus providing the basis for “imaging” the Earth’s magnetic field. However, it is unclear whether the property of entanglement is important for this function (66).

More generally, there are obvious major challenges for proving that the fundamental quantum correlations coming from entanglement underpin function. First, how can entanglement be measured in complex systems? It is very difficult to perform experiments to expose entanglement, as is well known. Moreover, it is exponentially difficult to accomplish such an analysis as the system size becomes larger. It therefore seems that systems exploiting entanglement in biology must be based on simple components. That is, they comprise a protein pocket in which two spins can separate and interact, like in the avian navigation example.

Second, how would molecular-scale function enabled by entanglement scale up to the biological scale—and still retain distinct signatures of quantum correlations? It is possible for a signal initiated on the molecular scale to be amplified; vision serves as an example. The challenge we wish to emphasize is that we might need to work backward from the amplified signal to ascertain that the function began through a quantum-mechanical mechanism.

Third, quantum correlations are often accompanied by classical correlations. For example, quantum interference phenomena are distinguished from classical interference only by subtle second-order statistics of the constituent quantum particles (67). To what extent does a function depend on uniquely quantum correlations, therefore, might be difficult to evaluate. Moreover, perhaps it is not essential to make the distinction.

### Probabilities that Appear to be Quantum-Like.

In the quantum world, probabilities are replaced with probability amplitudes, meaning they can be negative (or complex). Probabilistic outcomes can therefore be strongly modified through interference between alternative “pathways” (37). The probability amplitudes can be thought of as waves, such in-phase waves interfere constructively, and out-of-phase waves interfere destructively. The probability is calculated as the modulus squared of the net amplitude, which greatly enhances the effect.

Interestingly, psychology provides examples of experiments that appear best interpreted by postulating some kind of interference effect in the way the mind works. For instance, there is an accumulation of evidence that typical decision-making does not follow the usual rules of classical probability theory. Instead, the mind somehow ranks lists of joint information and weights certain conceptions, so they dominate or become integral in the decision or perception. A compelling approach for understanding these observations and explaining the prevalence of the conjunction fallacy has been to side-step Kolmogorov probability laws and, instead, use laws akin to those underpinning the quantum theory (68, 69).

Khrennikov has shown how these quantum-like probability laws incorporate interference effects because of the way context changes from, say, one question to the next (70). The quantum-like probability can therefore be written as a sum of the expectation from classical (Kolmogorov) probability laws and an interference term “correction” (71). Busemeyer and Pothos, in particular, have developed a detailed model for quantum cognition at the amplitude level based on a similar intuitive picture of information state vectors and projections (72–74). The crucial ingredient in these theories is that measurement—answering a question—has a back-action on the system. This back-action is a unitary transformation that changes the projection of an information state onto the answer to a subsequent question.

Here, we see an example of a classical phenomenon that is rationalized by a theory that is inspired by quantum mechanical probability laws. This cautions the quantum biologist—a model can suggest a quantum mechanical basis for function, but it is not proof of it. On the other hand, this example suggests that quantum biology may not require strict quantum mechanical systems supporting function, it may be sufficient—or even desirable, as we contend below—to mimic to some extent the desired quantum properties using classical machinery.

### Proposed Questions.

It is widely recognized that the field of quantum biology needs clear targets for the kinds of discoveries that will reveal how and why quantum phenomena can be engaged by living systems. For such examples to be influential and compelling, they should exhibit three characteristics: (i) A new function is enabled that is out of reach of a purely classical system; (ii) That new function should be biologically significant—it gives the organism an advantage; (iii) The mechanism underpinning the function is nonclassical in the sense that it is explained by quantum theory. We propose three open questions that direct the program for elucidating and comprehending examples of quantum biology.

Question 1: What measurements can reveal incisive evidence for quantum function?

A challenge in experimental design, that has been debated in particular with respect to studies of coherence in photosynthetic light-harvesting, is whether the experimental setup generates coherence that is not there in the natural operation of the system (75). A critical experimental issue in that field is that all the experiments use ultrashort light pulses which inevitably create coherences in the system under study. The system, even if excited incoherently, may generate its own coherences but the question “Did we do it or did the system do it?” has proved very difficult to answer definitively.

This example emphasizes, more generally, the need for the development of experiments that can detect quantum operations or functions obscured within complex biosystems. An abstract concept for an ideal experiment is to treat the system as a “black box” and design an experimental protocol that can detect or witness the system’s potential to exploit stronger-than-classical correlations.

A concrete possibility for such developments is demonstration of new quantum probes for biological systems (76, 77). A quantum probe is a second quantum system, prepared in a particular initial state, and used for an indirect measurement of the quantum system of interest (the primary quantum system). The probe interacts with the primary quantum system, then the new state of the quantum probe is measured in a second step of the measurement process (78). A specific example involves the quantum behavior of light. When the number of photons is small their true quantum nature is revealed in higher-order correlation functions (67). The change in photon statistics from excitation source to, for example, high time resolution emission detection of the second-order correlation function *g*^(2)^(*t*) of sample emission, has the potential to directly reveal coherent dynamics in systems that interact with light (79).

What sort of advantages could a quantum probe bring to photosynthetic light harvesting for example? In a large chromophore array, with an inhomogeneous distribution of energy levels, if the excitation moves as a wave (an exciton), the exciton is less likely to be trapped in a lower energy state far from the reaction center (80). If the exciton can reach the system’s lowest state (acceptor) by more than one pathway wavelike motion will allow constructive or destructive interference on the acceptor (80, 81). Whether the interference is constructive or destructive could be altered by very small structural changes produced allosterically, for example. By observing emission from the acceptor, these types of interference effects may be manifest in the structure of *g*^(2)^(*t*) (79). Experiments on photosynthetic systems with lower time resolution for *g*^(2)^(*t*) have been carried out (82, 83). These studies have shown that a single photon is sufficient to initiate transfer to a well-separated acceptor and by extension to the photosynthetic reaction center (82). In related experiments the fluorescence quantum yield and fluorescence lifetime of B850 emission did not depend on whether excitation was by single photons or a pseudothermal light source, while the very different photon statistics of the two sources were replicated in the emission statistics (83).

Further examples of quantum probes for quantum sensing are nitrogen vacancy centers in diamond. These materials allow various probe modalities in biological systems (84) and are seen to be especially attractive for enabling molecular-scale magnetic resonance measurements (85, 86). A great deal of work in this area suggests that developing various quantum sensors sensitive to spins or local magnetic fields is practical and a direction well-suited for applications in quantum biology. Steps in this direction are already underway. For example, new work shows that magneto-responsive fluorescent proteins can be expressed in living cells and used to probe local cellular environments (87). Superradiance has been considered as a witness for entanglement of electronic excitations (88–90).

Developing the application of quantum probes for quantum phenomena in complex systems will likely require development of new kinds of probes and measurement techniques. Developments in this direction will be important for identifying verifiably quantum effects.

Question 2: Do there exist complex classical systems possessing states that convincingly mimic useful properties of quantum states, thus giving the classical system an apparent “quantum advantage”?

Some of the most inspiring manifestations speculated for quantum biology involve very large and very complex systems, with associated complex functions. Quantum function connected to neural networks or brain function is one concrete example. As pointed out by Roger Penrose, “…we must seek quantum systems that retain their quantum nature on incredible scales of size and complexity.” (91). The difficulty is that we expect decoherence to dominate in these kinds of large and complex “messy” systems, as was famously noted by Tegmark (92). On the other hand, if some kind of quantum attribute on a biological scale makes a difference for an organism, it is likely that evolution would have led to a solution.

This idea leads us to ask: How would nature solve the decoherence problem? Well, imagine that we can construct a huge, complex classical system that mimics the separable states of a quantum system. Then we can engage the power of synchronization phenomena to maintain coherence, which is not possible in purely quantum systems because states evolve according to linear maps (quantum mechanics is linear rather than nonlinear). To understand synchronization in this context, consider a few people out strolling. We each walk at the speed and direction that takes our fancy. But on a city sidewalk in rush hour, crowds travel as a cohesive unit—we are synchronized. Synchronization is ubiquitous and powerful (39); it is the opposite of decoherence, the prevalent process that erodes fragile quantum states. However, synchronization is generally dismissed as a resource for quantum technology because synchronization is nonlinear, whereas the evolution of quantum states is linear. However, this powerful principle can be utilized by a classical system that emulates a quantum system.

Generally, a classical system does not mimic a quantum system. The question, though, is whether such classical systems exist at all? To answer that question we essentially need a strategy for looking for a needle in a haystack, because we expect an infinitesimal fraction of classical systems to possess the properties we need. How can we sort through all those classical systems? We need a kind of guide, or “Rosetta Stone,” that shows how a suitable classical system must be configured for it to be associated to a state space that is quantum-like. In recent work, we have developed (93–95) the required “Rosetta Stone” using the mathematics of graph theory (96).

The states of these special networks (graphs) mimic the abstract structure underlying the product functions that are the basis for states of quantum systems. Moreover, contrasting the paradigm that quantum states are fragile, this new principle allows states with “quantum-like” properties to thrive in highly complex systems, regardless of noise and disorder. The underlying networks are constructed using the powerful mathematics of expander graphs (97). These striking entities are guaranteed to produce a single distinguished state that serves as a starting point for the construction of quantum-like states (98). Quantum-like (QL) states are generated by binding expander graphs in a special way. The main advance is to “multiply” two or more quantum-like state graphs in such a way that the spectrum comprises an emergent superposition of tensor product states (94). All of this can apparently be carried out by a classical system. A classical system cannot produce entanglement, but the coherent QL separable states can nevertheless be a valuable resource.

In sum, if we think like biology and its recourse to 3.5 billion years of evolution, we conclude that functional biological systems that exploit a quantum advantage must be robust. That reasoning suggests that a living system striving for a very complex quantum-inspired function would likely mimic a quantum system rather than incorporate a genuine quantum system. Recent work suggests the existence of such classical systems. The conjecture posed here addresses one of the most pressing issues holding back the field of quantum biology: How can quantum effects be present in large, interesting classical systems where we normally expect decoherence to quench quantum coherence?

A connected open question is: How do we go about looking for such a system? Where would we find it in biology? The obvious answer is that the neural networks in the brain are sufficiently complex that they could feasibly incorporate a QL topology. Evidence might be revealed by functional MRI methods. Whether or not QL networks are useful depends on a second open question: How would the special correlations embedded in these structures be exploited for a functional advantage?

Question 3: How are the microscale mechanisms that engage quantum-mechanical or quantum-like phenomena amplified to the biological scale so as to provide a functional basis for new classical functions?

Many of the examples and ideas discussed in this paper posit that effects on the quantum scale may be obscured when we perform experiments on the bioscale. This rescaling of a quantum function to the biological scale amplifies the function to a classical system, analogous to the way a small periodic signal is imaged to a large stochastic signal in stochastic resonance. Fundamental identification of these kinds of amplifications of quantum effects is of interest for quantum biology, but we need a platform for thinking about the issue generally.

A recent paper explores the idea that an analogy to the concept of software versus hardware in computer science might clarify how to analyze complex emergent phenomena in nature (99). A key idea is that software can be quite separate from the hardware it runs on. The paper explores how that concept could be manifest. One principal proposal is that biological machinery can be partitioned by “information closure” that divides a natural system into interacting components. A secondary idea is that the details and inherent complexity at the microscopic scale are blurred at the mesoscopic scale by coarse-graining. Taken together, we can rationalize in an operational manner how, say, the vascular system can be understood entirely adequately without needing to know details about the “hardware”: various cells in the heart, veins, blood, etc.

The implication of this kind of coarse-graining that partitions a system into hardware and software, is that we view function though the software. But the microscale systems, and any quantum mechanical components, are likely embedded in the hardware. This viewpoint further emphasizes how the quantum in quantum biology can be hidden and, again, emphasizes the need for specially designed experiments to examine this question.

An example is the process of vision. Normally we consider the average macroscopic outcome of images on the retina translated by our brain. However, the detection apparatus has remarkable single-photon sensitivity (100), meaning that at the hardware level, vision works at the quantum scale. Question 3 could be addressed by exhibiting examples of how quantum phenomena are amplified to a macroscale function. What would be especially compelling would be an example where the macroscale function is different than could be achieved with an underlying classical, rather than quantum, system.

## Conclusion: What is Quantum Biology?

In this article, we have presented quantum biology as the idea that, if there is an advantageous function accessible in the quantum world, living systems have likely found a counterpart that is suited for a biological setting. A challenge is that many of these effects may be obscured because they are amplified into classical function.

We discussed several areas of relevance, concluding that the most concrete areas that comprise quantum biology are: i) The interaction between biological machinery and fields—light, magnetic, electric fields, and so on; ii) The many different kinds of coherence phenomena; iii) Proteins incorporating microscale quantum-dynamical systems that include spins or proton tunneling; iv) The potential for very complex biological systems to mimic quantum phenomena by “hard-wiring” the exponential complexity into robust networks.

The concept of quantum biology has persisted since the advent of the quantum mechanical theory, but it remains somewhat out of the mainstream. The field needs a compelling and simple example of function that would not normally be found in a classical system—but such a discovery has remained tantalizingly out of reach. Posing and discovering concrete and compelling examples of quantum biology is crucial. In a report developed during a recent workshop on “Optimal Coherence in Chemical and Biophysical Dynamics,” we discussed how to demonstrate that new quantum-relevant function can come from coherence in complex systems (37). We realized that coherence phenomena are prevalent in complex systems, but we failed to identify such systems and how they could serve as a resource for function. There is good reason to believe that advances in experimental strategies, informed by considering how quantum, or quantum-like, phenomena might hide in biological systems, will soon discover clear examples of how biology can engage the quantum world.

## Data Availability

There are no data underlying this work.
